# Clinical Outcomes of Patients with Rare and Heavily Pretreated Solid Tumors Treated according to the Results of Tumor Molecular Profiling

**DOI:** 10.1155/2016/4627214

**Published:** 2016-07-21

**Authors:** Andrew Dean, Aisling Byrne, Mira Marinova, Ingrid Hayden

**Affiliations:** St John of God Hospital, Subiaco, WA 6008, Australia

## Abstract

Patients with heavily pretreated advanced cancer or with rare tumors are difficult to treat. Molecular profiling (MP) of tumors to identify biomarkers that predict potential outcomes with individual therapies is an emerging strategy to guide treatment decisions. Patients with rare tumors for which standard-of-care therapy was unavailable or more common tumors for which standard-of-care options had been exhausted underwent MP at a single Australian center. Data regarding treating physicians' choice of therapy, MP results and recommendations, and patient outcomes were collected. Seven patients had received prior standard first-line therapy (PST), 16 had rare tumors, and 31 had been heavily pretreated (HPT; ≥2 prior lines). Most treatments suggested by MP (541/594; 91.1%) were common chemotherapy drugs available in generic formulations. MP-guided therapy recommendations differed from physician's recommendations in 48 patients (88.9%). MP-guided therapy produced clinical benefit (improved QOL and/or performance status, symptoms, bodyweight, or RECIST) in 19/31 (61.3%), 11/16 (68.8%), and 3/7 (42.9%) patients with HPTs, rare tumors, and PSTs, respectively, and had a PFS ratio ≥1.3 in 22/37 evaluable patients (59.5%; 95% confidence interval 44–76%). The null hypothesis that ≤15% of these patients would have a PFS ratio ≥1.3 was rejected (one-sided *p* < 0.0001). In conclusion, using MP to guide therapy selection is feasible in clinical practice and may improve patient outcomes.

## 1. Introduction

Good performance status patients with heavily pretreated tumors and those with rare malignancies represent a difficult therapeutic group. The aim of therapy for these patients is to extend survival while maintaining the best possible quality of life (QOL); however, balancing the risks and benefits of treatment that is often not evidence based represents a significant challenge [1, 2].

Traditionally, cancer treatment selection has been based on tumor organ of origin and histological type rather than tumor molecular characteristics, despite increasing genetic heterogeneity as tumors metastasize, suggesting a hypothesis for why only a proportion of patients respond [3]. As our understanding of tumor biology has improved, increasing numbers of factors predicting sensitivity to therapy have been identified and can be used to guide therapy, with potential utility for both new targeted/biological agents and chemotherapy [4]. For example, the epidermal growth factor receptor- (EGFR-) targeted agent cetuximab was initially developed for colorectal cancers overexpressing EGFR, but subsequent analyses have demonstrated that* KRAS* wild-type and* NRAS* wild-type status identify those tumors that are most sensitive to cetuximab [5], resulting in a new standard molecular profiling test to guide treatment selection. Similarly, molecular testing can result in new treatment options becoming available; for example, EGFR2- (HER2-) directed therapies are now recommended by the National Comprehensive Cancer Network for the treatment of those relatively rare lung cancers that harbor HER2 mutations [6, 7].

Molecular profiling (MP) using comprehensive screening for multiple tumor biomarkers has the potential to identify therapies to which a tumor is most likely to be sensitive or resistant. By combining this information with the treating physician's knowledge of anticancer drug regimens and patient history, potentially effective regimens can be identified. Use of this technique in a variety of tumor types has been reported [8–13].

We describe a review of prospectively collected data for patients with difficult-to-treat tumors from a single center who underwent MP with a view to using the results to guide treatment decisions.

## 2. Materials and Methods

### 2.1. Patients

A single practice at St John of God Hospital, Subiaco, Western Australia, offered MP to a consecutive series of good performance status patients with rare tumors with limited or no standard treatment options available and to those with common tumors who had exhausted standard treatment options. Patients who had received prior standard first-line therapy (PST) could request MP. All patients who underwent MP were included in the study, but those who died prior to receiving treatment or who subsequently opted out of MP-guided treatment were excluded from the efficacy analysis.

### 2.2. Molecular Profiling

MP was performed using the Caris Molecular Intelligence*™* (CMI) platform. Using this platform, formalin-fixed paraffin-embedded tumor specimens were analyzed for multiple biomarkers using techniques such as immunohistochemistry, fluorescence/chromogenic* in situ* hybridization, quantitative polymerase chain reaction, and direct gene sequencing. Sample analysis is typically completed within 14 days. All biomarkers tested for are included in the panel based on the strength of supporting evidence as defined by the United States Preventative Services Task Force (USPSTF) level of evidence methodology [14]. The biomarker results are then interpreted to determine which of a panel of therapies is likely to provide benefit based on published evidence (>95% of the associations included are supported by level 1 or level 2 evidence). The biomarker panel is updated based on ongoing literature review, meaning that the precise number of biomarkers analyzed for an individual patient varied.

### 2.3. Therapy Selection

Each patient had a “best unprofiled treatment choice” documented. However, the patient was treated based on the results of subsequent MP, with final treatment decisions being made based on a number of considerations. First, patients who had previously progressed on a drug that was identified as “likely to provide benefit” by MP were not retreated with the same drug because prior progression implies that other intracellular resistance pathways have become dominant. Second, where a number of different drugs were identified by MP as potentially beneficial, standard combination regimens were identified by the investigators and used (e.g., cisplatin plus gemcitabine; irinotecan plus 5-FU). Third, if there were reasons to exclude a particular drug or drug class (e.g., known severe hypersensitivity or prior intolerability), a regimen with less risk was chosen. Finally, where several drugs showed potential benefit and the factors above were unable to select between them, those drugs considered likely to provide most benefit by the investigators were selected.

### 2.4. Assessments

Treatment benefit was assessed after each treatment cycle based on factors including symptom relief and changes in bodyweight, pain, performance status, tumor marker levels, and patient-reported QOL. Tumors were imaged every 2–4 cycles. Response to treatment based on imaging was evaluated using Response Evaluation Criteria in Solid Tumors (RECIST) [15]. Progression-free survival (PFS) was defined as the time from the MP request until Eastern Cooperative Oncology Group (ECOG) performance status deterioration (assessed at each cycle of therapy) or progression as defined using RECIST [16].

To assess the benefit of MP-guided therapy, we used a previously described technique in which patients act as their own controls [13] by assessing the ratio between PFS on MP-guided therapy and that on the most recent prior therapy. Von Hoff et al. defined a ratio of ≥1.3 as being indicative of clinical benefit with MP-guided therapy [13].

### 2.5. Statistical Analysis

A one-sample one-sided proportion test was performed to test the null hypothesis that ≤15% of the patients would have a PFS ratio ≥1.3. This approach was based on that used in the previous study reported by Von Hoff et al. [13], which assumed a null response rate of 15% and an alternative response rate of 30%. These assumptions were used here. Considering these, the known sample size (*n* = 37 with known PFS ratio), and an *α* risk of 5%, the power of the statistical test was 75%.

## 3. Results

### 3.1. MP Findings

**Figure 1:**
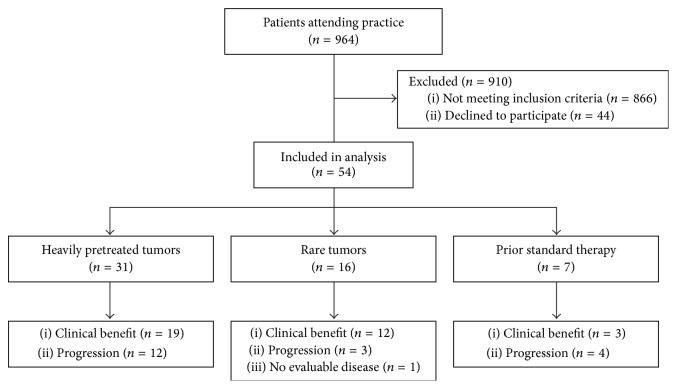
Flow of patients in the study.

Patient disposition is shown in Figure 1. Between March 5, 2012, and March 11, 2013, 98 consecutive patients who met the inclusion criteria were offered MP, with 54 patients undergoing MP and being treated according to the profile (Tables 1 [Table tab2]–3): 31 had heavily pretreated tumors (HPT; ≥2 prior lines); 16 had rare tumors; and 7 had received PST. Patients in the HPT and rare tumor groups had received a median of 2 (ranges 2–4) and 1 (ranges 0–2) prior lines of therapy, respectively. MP identified a median of 18 (range 8–26) biomarkers associated with drugs with likely benefit (median 8; range 2–15) or lack of benefit (median 8; range 2–19) per patient in the HPT group, 16 (range 10–23; with benefit 7.5 [3–13], without benefit 9 [2–16]) per patient in the rare tumor group, and 13 (range 9–22; with benefit 5 [3–7], without benefit 10 [3–15]) per patient in the PST group. The proportion of biomarkers with benefit appeared to be greater in the HPT (47.6%) and rare tumor groups (45.3%) than in the PST group (36.9%).

The majority of agents suggested by MP (541/594; 91.1%) were widely available chemotherapy drugs. Targeted/biological therapy recommendations were made predominantly for patients in the HPT group (45 of 53 targeted/biological agents recommended); reflecting this, the majority of the 16 patients in whom novel targeted/biological therapy recommendations were made based on MP were in the HPT group (13 patients). The next best MP-guided therapy recommendation was the same as the physician's recommendation in only six patients overall (11.1%).

### 3.2. Treatment Outcomes

Clinical benefit (improved QOL and/or performance status, symptoms, bodyweight, or RECIST) was observed in 19/31 (61.3%), 11/16 (68.8%), and 3/7 (42.9%) patients in the HPT, rare tumor, and PST groups, respectively (Tables 1 [Table tab2]–3).

**Figure 2:**
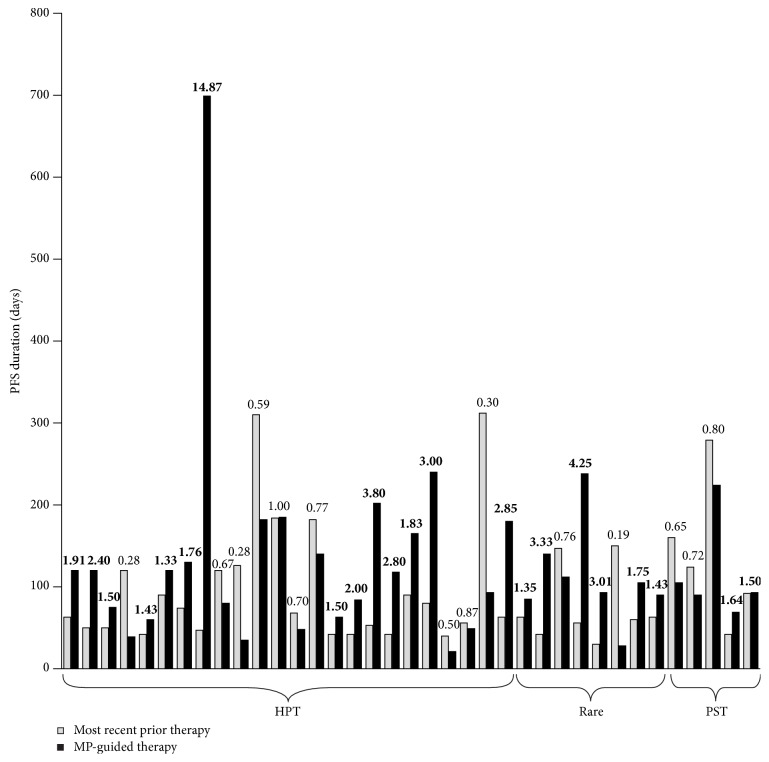
PFS with therapy suggested by MP versus that with prior line of therapy. The ratio between these two values is also shown.

Data on the PFS ratio were available for 37 of 54 patients (HPT, 24/31; rare tumors, 8/16; PST, 5/7) (Tables 1 [Table tab2]–3; Figure 2). Of these 37 patients, 22 (59.5%) had a PFS ratio ≥1.3 (HPT, 14/24 [58.3%]; rare tumors, 6/8 [75.0%]; PST, 2/5 [40.0%]) (95% confidence interval: 44–76%). The null hypothesis that ≤15% of these patients would have a PFS ratio ≥1.3 was rejected (one-sided *p* < 0.0001). The median PFS ratio was 1.75 for those categorized as having a response and 0.80 for those who did not have a response.

Although targeted/biological therapies were recommended by MP in 16 patients, only four patients were treated with targeted/biological therapy. Three of the 13 patients in the HPT group in whom targeted/biological therapy was identified as being of potential benefit by MP received it: one patient with heavily pretreated leiomyosarcoma that responded to MP-guided sunitinib (PFS ratio 14.87); one patient with metastatic breast cancer that responded to trastuzumab combined with irinotecan (PFS ratio 0.67); and one patient with pancreatic adenocarcinoma that responded rapidly to trastuzumab + erlotinib after failing MP-guided epirubicin + cisplatin + 5-FU (ECF) (PFS ratio 1.33). One patient with a PST tumor (small-cell lung cancer) received targeted/biological therapy (cetuximab + FOLFIRI), without apparent benefit; another, also with small-cell lung cancer, in whom targeted/biological therapy was suggested but not used, showed a RECIST response and stable QOL and performance status with MP-guided FOLFIRI (PFS ratio 0.80). In most of the other cases, the recommended targeted/biological therapy was either not funded or not available and MP-guided chemotherapy was used instead.

Individual patients showing noteworthy responses included three in the HPT group, two with significant shrinkage on imaging of cervical tumors treated with MP-guided irinotecan + 5-FU (PFS ratios 1.91 and 2.00), and a promising response to gemcitabine + nab-paclitaxel (stable disease with improved QOL, performance status, and symptoms) in a patient with mesothelioma (PFS ratio 0.30; survival 7.0 months). All three patients showed improved performance status and experienced clinically significant pain relief. In the rare tumor group, a patient with anaplastic thyroid disease who had previously had no treatment options had notable survival (7.1 months) after second-line FOLFIRI treatment; another patient with adrenal cortex carcinoma showed RECIST response to FOLFIRI after failure of two previous lines of therapy (PFS ratio 3.33).

## 4. Discussion

This observational study suggests utility of using MP to select treatment in patients with tumors for which treatment options are limited or otherwise exhausted. MP-guided treatment matched the clinician's unguided choice of next best treatment in only 11% of cases, indicating the difficulty of selecting appropriate treatment for these patients. Thus, the observation that approximately 60% (95% confidence interval 44–76%, *p* < 0.0001) of patients obtained clinical benefit from MP-guided therapy, whether based on investigator-assessed response or PFS ratio, is particularly noteworthy.

Moreover, the majority of treatments suggested by MP were standard chemotherapeutic drugs as opposed to novel targeted/biological agents. Thus, MP appears to provide rational, evidence-based treatment selections often based on standard chemotherapy regimens, with no apparent bias toward novel targeted/biological therapy. Individual data revealed significant and unexpected benefit in a number of patients with particularly aggressive types of tumor. This suggests that MP could be a valuable aid to clinical decision-making in patients with advanced tumors who have exhausted the available standard therapeutic approaches. The ability of MP to target the use of standard chemotherapies to those patients most likely to benefit, as well as avoiding unnecessary treatment, is a significant potential advantage.

Although our data are observational and the statistical analysis that could be performed was limited, we noted an overall link between apparent clinical benefit and increases in PFS ratios. The PFS ratio is a new endpoint that is still debated in the literature [17] and may be vulnerable to ascertainment bias [14] but has been shown to give an indication of treatment effect in clinical studies where patients act as their own controls [13, 18, 19]. Our observations concur with these results.

Increased understanding of the molecular pathways involved in malignant disease, coupled with developments in tumor molecular analysis, has stimulated interest in identifying ways of further optimizing the use of targeted agents through successfully identifying those patients most likely to respond. MP offers an opportunity to use conventional chemotherapy in a targeted manner.

It has long been recognized that identifying those patients most likely to respond is more complicated than assessing tumor expression of the therapeutic target, with other tumor molecular changes most likely to explain why not all patients respond to a targeted drug despite target expression. Subsequent investigations have shown that selecting therapy by considering tumor aberrations other than the specific target has the ability to predict response and PFS [8] and that MP is associated with clinical benefit and increased PFS ratios in patients with refractory metastatic tumors [13].

Patients with refractory metastatic tumors present a particular challenge. These are the patients usually enrolled into phase I trials; the need for improved treatment options for these patients is illustrated by the fact that response rates in phase I trials are often around 10% whereas the response rate for the general population of patients with cancer is approximately 35% [2, 20]. In addition, patients with advanced cancer suffer severe physical and psychological symptoms, and symptom control and maintenance of QOL are key considerations [21]. Clinicians' estimates of clinical benefit and patient-reported QOL in this study suggest that MP-guided therapy may also assist palliative efforts in these individuals.

These data are subject to a number of limitations, mainly due to their observational nature and the limited statistical analysis that could be performed. No randomization was used, with each patient acting as their own control. Thus, the results obtained with MP-guided therapy could not be compared with the physician's initial choice of next best therapy. In addition, patients had a broad range of tumor types and much information was recorded descriptively. Nevertheless, as a representative series of difficult-to-treat patients, our data indicate the potential of MP in guiding therapy, supporting the findings of others such as Tsimberidou et al. [8].

Future studies should assess the feasibility of randomized trials comparing MP-guided with the treating physician's choice of treatment in disease-specific settings using consistent and objective endpoint measures and validated QOL and pain instruments. However, designing trials to effectively assess MP will be complex, due primarily to tumor heterogeneity; for example, Schwaederle et al. reported that no two tumors examined by MP in a series of 34 patients had the same aberrations [22]. Therefore, randomized trials may not be optimal [23]. Furthermore, endpoints that are sensitive enough to identify differences in outcomes in heterogeneous populations will be needed [23]. In addition, although studies suggest that the use of biomarkers in guiding treatment may be cost effective or even cost saving [24, 25], further investigation is needed because the use of MP may currently be restricted by concerns regarding cost [26].

## 5. Conclusion

This study suggests that the use of MP in clinical practice to guide treatment decisions in patients with difficult-to-treat tumors is feasible and appears to produce favorable outcomes.

## Figures and Tables

**Table 1:** Tumor type and clinician and MP-guided therapy recommendation, MP results, and clinical outcomes: heavily pretreated group (HPT).

Tumor location	Prior lines	Next best treatment: clinician-defined	Next best treatment: MP-defined	Clinical observations^a^	Biomarkers with/without benefit	Chemotherapy versus biological/targeted therapy	PFS ratio^b^
Cervical	2		Irinotecan + 5-FU (FOLFIRI started)	CB	5/5	7 versus 2	**1.91**
Gall bladder	3		FOLFIRI	CB	3/5	5 versus 0	**2.40**
Skin (Merckel)	3		Epirubicin + cisplatin + 5-FU	P	4/5	8 versus 0	**1.50**
Colorectal	3	Mitoxantrone/5-FU	Epirubicin + cisplatin + 5-FU	P	4/5	8 versus 0	0.28
Cervical	2	Nil	FOLFIRI	CB	15/4	10 versus 7	**1.43**
Pancreas (adenocarcinoma)	2	Nil	Epirubicin + cisplatin + 5-FU then trastuzumab (?)/erlotinib	CB	7/2	7 versus 6	**1.33**
Gastric	2	FOLFIRI	Gemcitabine + oxaliplatin	CB	15/5	12 versus 3	**1.76**
Leiomyosarcoma	2	Trabectedin	Sunitinib	CB	11/6	9 versus 7	**14.87**
Breast (ER^−^/HER2^+^)	3	Trastuzumab/capecitabine then liposomal doxorubicin then trastuzumab/irinotecan	Trastuzumab + irinotecan	CB	15/9	20 versus 3	0.67
Uterus	2		Doxorubicin + cyclophosphamide	P	6/16	8 versus 0	0.28
Breast	2		Nab-paclitaxel (intolerant), cyclophosphamide + methotrexate + 5-FU	P	8/11	17 versus 0	NA
Small bowel	2		Died before assessment	P	6/16	7 versus 0	NA
Esophageal	2		FOLFIRI	P	7/16	7 versus 2	NA
Lung	2		Cisplatin + pemetrexed	CB	14/12	7 versus 3	0.59
Malignant mesothelioma	2	Cisplatin + pemetrexed	Cisplatin + nab-paclitaxel + doxorubicin	P	5/19	4 versus 0	NA
Ovarian	3	Nab-paclitaxel	Topotecan (also anthracyclines/nab-paclitaxel/hormones)	CB	8/15	12 versus 0	1.00
Cholangiocarcinoma	2	Epirubicin + cisplatin + 5-FU	Cisplatin + gemcitabine	CB	5/13	7 versus 0	NA
Mesothelioma	2	Nil	FOLFIRI	P	2/16	5 versus 0	0.70
Gastrointestinal	2	FOLFIRI	FOLFIRI	P	4/18	5 versus 0	0.77
Colon (adenocarcinoma)	2	Mitomycin-C + 5-FU	Carboplatin + gemcitabine	CB	13/8	10 versus 2	**1.50**
Cervical	2		Irinotecan + 5-FU (FOLFIRI started)	CB	5/5	7 versus 2	**2.00**
Ovarian (serous adenocarcinoma)	2	Cisplatin + liposomal doxorubicin	Carboplatin + liposomal doxorubicin	CB	9/9	14 versus 0	**3.80**
Lung (adenocarcinoma)	3	Vinorelbine	Nab-paclitaxel	P	10/11	9 versus 0	NA
Pleura (adenocarcinoma)	3		FOLFIRI	CB	9/6	11 versus 0	**2.80**
Prostate	3	Mitoxantrone	Irinotecan + 5-FU	CB	12/5	16 versus 0	**1.83**
Ovarian (serous adenocarcinoma)	2	Cisplatin + liposomal doxorubicin	Cisplatin + liposomal doxorubicin	CB	9/4	10 versus 0	**3.00**
Ovarian (serous adenocarcinoma)	2	Cisplatin + liposomal doxorubicin	Doxorubicin	P	6/8	7 versus 2	0.50
Colon (adenocarcinoma)	2		Gemcitabine + nab-paclitaxel	P	8/9	8 versus 0	0.87
Mesothelioma	2	Vinorelbine	Gemcitabine + nab-paclitaxel	CB	8/7	7 versus 0	0.30
Gastric (adenocarcinoma)	2	Etoposide + leucovorin + 5-FU	FOLFIRI	CB	10/6	11 versus 3	**2.85**
Ductal breast carcinoma	1		Nab-paclitaxel	CB	10/3	16 versus 3	NA

*Totals*				*CB = 19; P = 12*	*253/279*	*291 versus 45*	

^a^CB = clinical benefit (QOL/PS/general symptoms/weight and imaging results improved or stable); NA = not available; P = progression (QOL/PS/general symptoms/weight and imaging results declined or deteriorated).

^b^Ratio of PFS with therapy suggested by MP versus that with prior line of therapy.

FOLFIRI, irinotecan + 5-FU + folinic acid; PFS, progression-free survival; PS, performance status; QOL, quality of life.

**Table 2 tab2:** Tumor type and clinician and MP-guided therapy recommendation, MP results, and clinical outcomes: rare tumor group.

Tumor location	Prior lines	Next best treatment: clinician-defined	Next best treatment: MP-defined	Clinical observations^a^	Biomarkers with/without benefit	Chemotherapy versus biological/targeted therapy	PFS ratio^b^
Ethmoid sinus	1		Pemetrexed	CB	7/7	3 versus 6	**1.35**
Adrenal cortex (adenocarcinoma)	2		FOLFIRI	CB	6/15	16 versus 0	**3.33**
Thyroid (anaplastic)	1	Nil	FOLFIRI	CB	7/3	12 versus 0	0.76
Fibrosarcoma	0		Anthracyclines + dacarbazine (MAID)	CB	7/16	16 versus 0	NA
Astroblastoma	2	Temozolomide	Temozolomide	CB	4/9	5 versus 0	NA
Pseudopapillary mucinous neoplasm	1	FOLFIRI	Cisplatin + 5-FU	CB	9/9	22 versus 0	**4.25**
Endometrial stromal sarcoma	1		Tamoxifen (also platinum therapy, 5-FU, irinotecan, gemcitabine, and nab-paclitaxel)	P	9/9	22 versus 0	NA
Medullary thyroid	0	Nil	Carboplatin + nab-paclitaxel	NED	3/19	7 versus 0	NA
Adrenal cortical carcinoma	1	Nil	Cyclophosphamide + doxorubicin + cisplatin	P	11/1	17 versus 0	NA
Liver leiomyosarcoma	2	Pazopanib	Gemcitabine + nab-paclitaxel then nab-paclitaxel only	CB	12/4	18 versus 0	**3.01**
Uterine carcinosarcoma	2	Cisplatin + liposomal doxorubicin	Cisplatin + liposomal doxorubicin	P	5/11	6 versus 0	0.19
Fibrolamellar hepatocellular carcinoma	1	Sorafenib	FOLFIRI	CB	8/7	9 versus 0	**1.75**
Carcinoma with unknown primary (mediastinum)	2		Cisplatin + nab-paclitaxel	CB	8/13	6 versus 0	**1.43**
Adenoid cystic carcinoma of the maxilla (metastatic)	1		FOLFIRI	CB	8/8	9 versus 0	NA
Carcinoma with unknown primary (sacrum)	2		Cisplatin + gemcitabine	CB	3/12	7 versus 0	NA
Uterine leiomyosarcoma	3	Pazopanib	Gemcitabine + nab-paclitaxel	CB	13/2	21 versus 0	NA

*Totals*				*CB = 11; P = 3; other = 2*	*120/145*	*196 versus 6*	

^a^CB = clinical benefit (QOL/PS/general symptoms/weight and imaging results improved or stable); NA = not available; NED = no evaluable disease; P = progression (QOL/PS/general symptoms/weight and imaging results declined or deteriorated).

^b^Ratio of PFS with therapy suggested by MP versus that with prior line of therapy.

FOLFIRI, irinotecan + 5-FU + folinic acid; MAID, mesna + doxorubicin + ifosfamide + dacarbazine; PFS, progression-free survival; PS, performance status; QOL, quality of life.

**Table 3:** Tumor type and clinician and MP-guided therapy recommendation, MP results, and clinical outcomes: prior standard first-line therapy group (PST).

Tumor location	Prior lines	Next best treatment: clinician-defined	Next best treatment: MP-defined	Clinical observations^a^	Biomarkers with/without benefit	Chemotherapy versus biological/targeted therapy	PFS ratio^b^
Lung	1	Docetaxel	Carboplatin + nab-paclitaxel	CB	5/6	4 versus 0	0.65
Pancreas (adenocarcinoma)	1	FOLFIRINOX	FOLFIRINOX	P	5/7	8 versus 0	NA
Melanoma	1		Fotemustine	P	7/14	7 versus 0	0.72
Lung (small cell carcinoma)	1		FOLFIRI	CB	5/10	8 versus 1	0.80
Gall bladder	1	Epirubicin + cisplatin + 5-FU	FOLFOX	P	3/10	7 versus 0	**1.64**
Lung (small cell carcinoma)	1	Docetaxel	Cetuximab + FOLFIRI	P	6/3	9 versus 1	NA
Gastrointestinal (adenocarcinoma)	1	Carboplatin + nab-paclitaxel	FOLFIRI	CB	7/15	11 versus 0	**1.5**

*Totals*				*CB = 3; P = 4*	*38/65*	*54 versus 2*	

^a^CB = clinical benefit (QOL/PS/general symptoms/weight and imaging results improved or stable); NA = not available; P = progression (QOL/PS/general symptoms/weight and imaging results declined or deteriorated).

^b^Ratio of PFS with therapy suggested by MP versus that with prior line of therapy.

FOLFIRI, irinotecan + 5-FU + folinic acid; FOLFOX, 5-FU + folinic acid + oxaliplatin; FOLFIRINOX, irinotecan + 5-FU + folinic acid + oxaliplatin; PFS, progression-free survival; PS, performance status; QOL, quality of life.
